# Mitigating the susceptibility to intergranular corrosion of alloy 625 by friction-stir welding

**DOI:** 10.1038/s41598-022-07473-0

**Published:** 2022-03-03

**Authors:** Guilherme Vieira Braga Lemos, Alexandre Bellegard Farina, Henrique Piaggio, Luciano Bergmann, Jane Zoppas Ferreira, Jorge Fernandez dos Santos, George Vander Voort, Afonso Reguly

**Affiliations:** 1 Programa de Pós Graduacão em Engenharia de Minas, Metalúrgica e de Materiais (PPGE3M), Universidade Federal Do Rio Grande Do Sul (UFRGS), Porto Alegre, Brazil; 2 Centro de Pesquisa e Desenvolvimento, Villares Metals S/A, Sumaré, São Paulo, Brazil; 3 Institute of Materials Mechanics, Solid State Materials Processing (WMP), Helmholtz-Zentrum Hereon, Geesthacht, Germany; 4 Vander Voort Consulting LLC, Wadsworth, IL USA

**Keywords:** Engineering, Materials science

## Abstract

In this work, friction-stir welding (FSW) was employed to alloy 625 grade I (soft annealed) sheets. Therefore, solid-state based welding was undertaken with a tool rotational speed of 200 rpm and a welding speed of 1 mm/s. Microstructural features were analyzed by light optical and scanning electron microscopy (LOM and SEM). Moreover, microhardness measurements were performed. The susceptibility to intergranular corrosion was verified by the double-loop electrochemical potentiokinetic reactivation (DL-EPR) test. Complementary, intergranular corrosion was also evaluated by the ASTM G28 Method A. FSW promoted grain refinement, increased microhardness, and reduction in the degree of sensitization. Finally, the mean corrosion rate observed in the ASTM G28 Method A test was 0.4406 mm/year, which suggests a good weld quality.

## Introduction

Alloy 625 (UNS N06625) is employed in the aerospace, nuclear, and oil and gas industries due to its high mechanical properties and exceptional corrosion resistance^1,2^. In these fields, joining methods for nickel alloys are usually based on fusion (arc welding), such as shielded metal arc welding (SMAW) and tungsten inert gas (TIG), amongst others. Nickel-based alloys are normally highly alloyed so that traditional fusion welding methods, heat treatments, among other manufacturing processes, can lead to formation of intergranular metal-rich carbides (metal often being Cr and Mo), Laves and Delta phases, and promote grain growth^1–3^, which tend to affect their corrosion and mechanical properties.

Alloy 625 sheets are typically supplied in two grades: soft annealed (grade I) and solution annealed (grade II), with differences in their properties. It is interesting to note that alloy 625 may be chosen for low or high temperature applications^4,5^. For example, grade I might have higher mechanical properties than grade II. Also, alloy 625 soft annealed has M(C, N) carbonitrides dispersed in the matrix as well as M_6_C and M_23_C_6_ carbides (normally rich in Mo and Cr) at some grain boundaries^2^. Moreover, although alloy 625 (also called Inconel 625®) has a solid-solution hardening mechanism; it is well known that carbide precipitation plays an important role for improving mechanical properties^6^. In consequence, nickel-based alloys may have increased mechanical properties due to carbides precipitation^7–9^. On the other hand, carbides can deteriorate the corrosion properties. In this context, when alloy 625 is typically exposed to a temperature range between 600 and 900 °C^10^, it could exhibit instability and sensitization. Therefore, the sensitization can be understood as the precipitation of metal-rich carbides preferentially at grain boundaries (area of the most free-energy^11^), which is a well-known phenomenon in stainless steels and aluminum alloys. Thus, the neighboring regions may become depleted of these metals and consequently more susceptible to intergranular corrosion.

Friction-stir welding (FSW) is a high-quality, solid-state joining method to overcome typical issues of the conventional joining techniques in nickel-based alloys because it produces sound joints at relatively low temperatures, but sufficient for recrystallization and small-sized grains to occur. Therefore, improved properties can be achieved by FSW^1^. In this process, a rotating tool promotes hot work, and the alloy is in the plasticized state for the weld processing. In general, the joint is processed autogenously with chemical composition of the base material^12^. However, FSW for high plasticizing and high melting point alloys is not well developed as it is worldwide recognized for aluminum alloys (low-melting point). In this context, a limited number of works have shown FSW in nickel-based alloys^1,2,9,12–16,23^, where pcBN tool wear is often reported. It remains unknown in investigating the intergranular corrosion properties of friction-stir-welded alloy 625, as indicated in^1^.

Intergranular corrosion of various alloys with a passive surface layer has been analyzed over time by distinct corrosion tests^17^. The first electrochemical tests were named after their inventors (Streicher, Strauss and Huey), as mentioned in^18^. Overall, if the investigated condition is sensitized, grain dropping would occur^19^. Aiming at substituting the long-term assays, the double-loop electrochemical potentiokinetic reactivation (DL-EPR) test was gratefully developed and accepted^18,20,21^. The main advantage of the DL-EPR test is to obtain fast results for the degree of sensitization (DOS). In this sense, nickel-based alloys have also been evaluated by DL-EPR, with some improvements in the test^17,22^. However, results of the DL-EPR tests in friction-stir-welded alloy 625 have not been found. Still, the intergranular corrosion using the ASTM G28 Method A remains to be explored in friction-stir-welded alloy 625, as proposed in the current investigation.

As intergranular corrosion is one of the most important degradation mechanisms of alloy 625^5^; it must also be evaluated in friction-stir welds of this nickel-based alloy. Therefore, the current work focuses on mitigating the susceptibility to intergranular corrosion of alloy 625 (grade I soft annealed) through the FSW process.

## Materials and methods

Friction-stir-welded alloy 625 sheets (300 mm × 80 mm × 3.2 mm) were investigated in this work, and their chemical composition is given in Table 1 (data from the supplier). Moreover, alloy 625 was in the soft annealed condition (grade I), which has exceptional corrosion resistance in varied corrosive media^23^. Also, this nickel-based alloy is often chosen for cladding rigid pipes due to its outstanding corrosion properties.

**Table 1:** Chemical composition of alloy 625 grade I (% by weight).

	Ni	Cr	Fe	Mo	Nb	Co	Mn	Al	Ti	Si	C
Alloy 625	Bal	21.7	4.7	8.6	3.38	0.03	0.09	0.13	0.18	0.18	0.015
Standard UNS N06625	> 58.0	20.0–23.0	< 5.0	8.0–10.0	3.15–4.15	< 1.0	< 0.5	< 0.4	< 0.4	< 0.5	< 0.1

Alloy 625 sheets were prepared by cleaning the surfaces by abrasive sanding and acetone, aiming at the removal of grease, oxides, and impurities. This step was recommended to increase the surface quality of the joint^2^. Afterwards, the sheets were fixed and aligned on the welding table. Welded joints were processed in the butt joint configuration on a rigid gantry machine, which has servomotors and automated control systems. A polycrystalline cubic Boron Nitride (pcBN) Q70 tool, with W–Re (binder phase), a shoulder of 25-mm diameter, and a probe of 3-mm long was used. The tool was tilted 1.5° to the vertical axis to obtain the welded joint. Also, welding was performed with an argon atmosphere to reduce oxidation. FSW was carried out with the best process condition (tool rotational speed of 200 rpm and welding speed of 1 mm/s), as done elsewhere^2,9,12,24^. These parameters can minimize wear of the pcBN tool. Details on pcBN tool wear in FSW of alloy 625, recently investigated by an experimental and numerical approach, can be found in^24^.

For microstructural analysis, the samples were cut by electrical-discharge machining (EDM), prepared by conventional metallography practices (sanding and polishing), and etched with glyceregia (10 ml HCl, 10 ml HNO_3_, and 0.5 ml glycerin). Thus, the microstructural features were observed by light optical microscopy (LOM) and scanning electron microscopy (SEM).

Local mechanical properties were evaluated in terms of microhardness. The Vickers microhardness profile measured on the top surface was obtained with a 500 gf load and distance between indentations of 0.2 mm.

**Figure 1:**
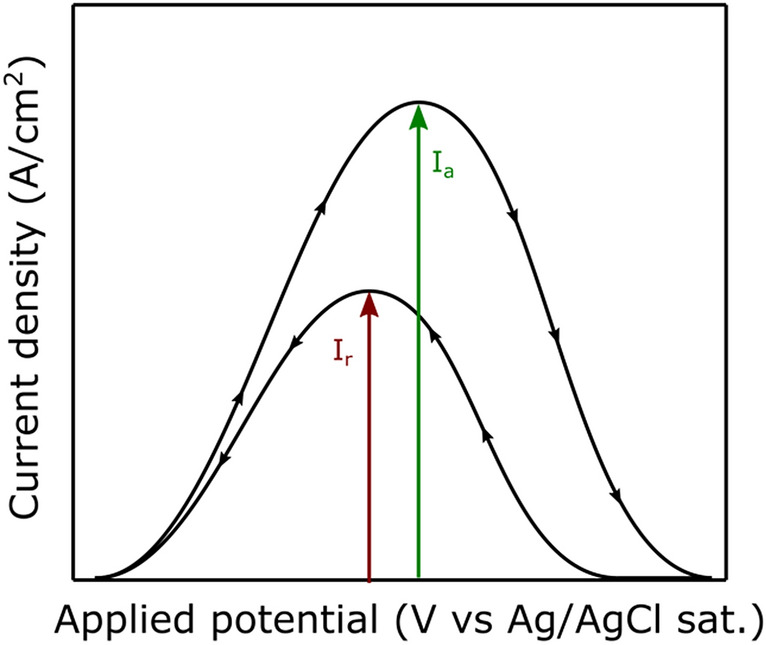
Schematic drawn of double-loop electrochemical potentiokinetic reactivation (DL-EPR) test.

The investigation of the degree of sensitization was based on the DL-EPR test, which is schematically shown in Fig. 1. The parameters of this electrochemical test were taken from^17^, where the regions chosen were firstly anodically polarized to the metal passivation zone and then scanned in the reverse direction. In other words, when a region is anodically polarized, it is theoretically covered by a passive layer. Next, when the scan is reversed, the passive film breaks in the metal-depleted areas. These metal-depleted areas (diminished Cr or Mo content) tend to be preferentially attacked due to anodic polarization effects that can create uneven surfaces^25^ or even a thinner and less protective oxide layer^26^, so that these act as preferential sites for the depassivation. Hence, the selected regions investigated were the base material (BM) and the stir zone (SZ).

The susceptibility to intergranular corrosion through weight loss was carried out in accordance with ASTM G28 Method A^27^. Two samples (12.5 × 50 × 3.2 mm) were machined by EDM, sanded and polished. The weight change was verified after the tests. A solution of 25 g FeSO_4_, 236 ml H_2_SO_4_, and 400 ml deionized water was used. The duration of the tests was 120 h (starting from the time that the solution reached the boiling point). Hence, the corrosion rate was calculated via Eq. (1), as follows:

1 $${\text{Corrosion rate}} = \frac{{{\text{constant}} \times {\text{weight loss}}}}{{{\text{area}} \times {\text{time}} \times {\text{density}}}}$$

where the constant was 86.760 and the density of alloy 625 was 8.44 g/cm^3^^5,27^. Furthermore, the samples tested were observed by LOM.

**Figure 2:**
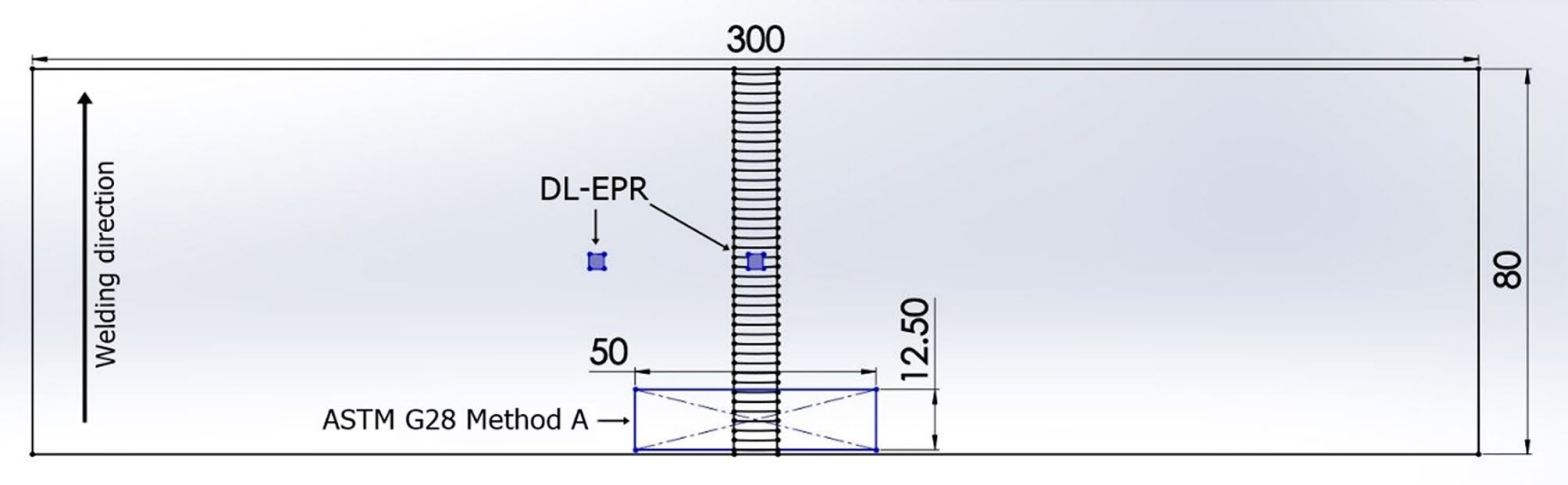
Schematic drawn showing the samples for corrosion tests (DL-EPR and ASTM G28 Method A).

A schematic sketch (Fig. 2) is therefore detailing the samples for both corrosion tests (DL-EPR and ASTM G28 Method A).

## Results and discussion

### Base material

**Figure 3:**
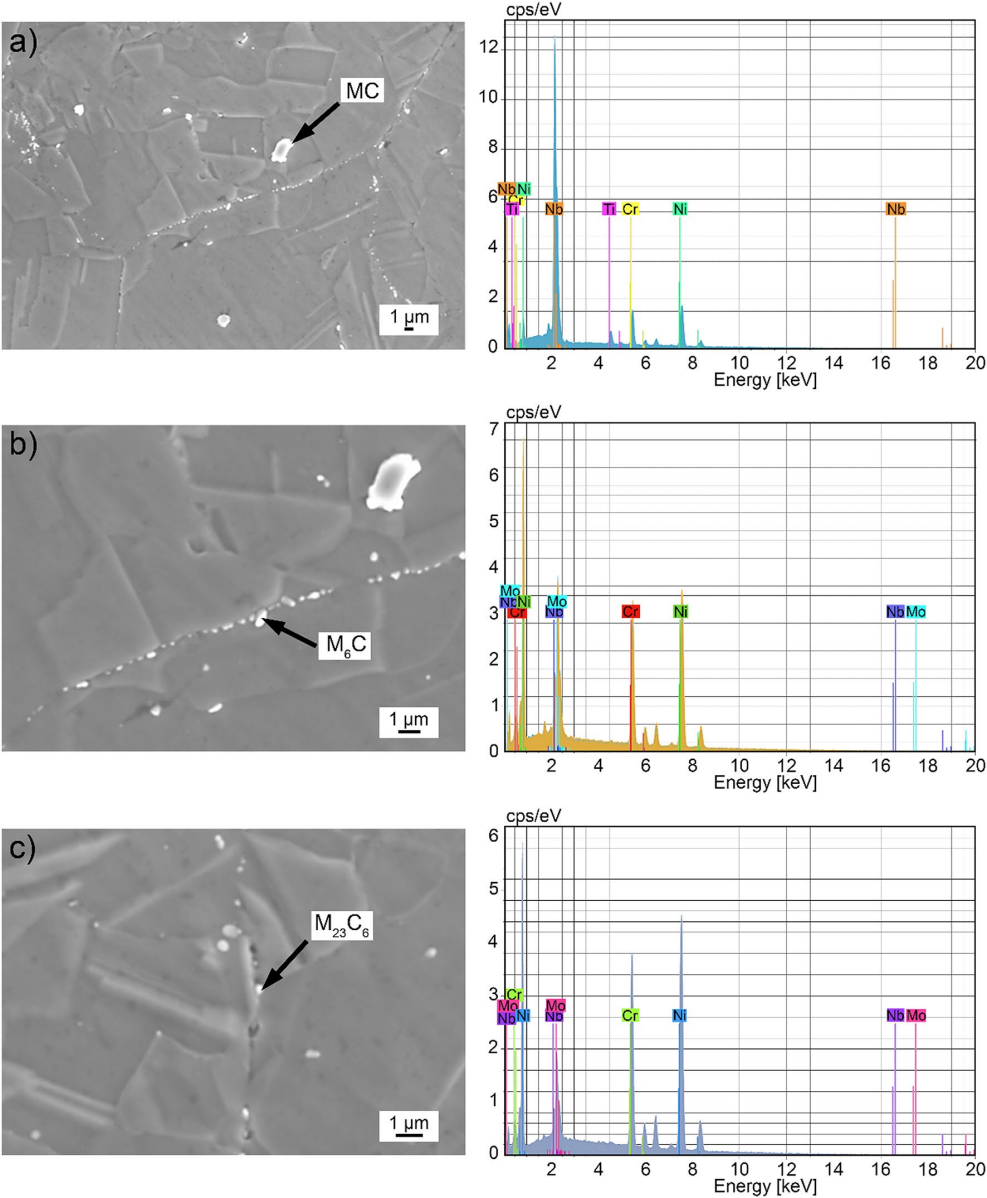
Base material microstructure (alloy 625 grade I soft annealed): (**a**) MC carbonitride, (**b**) M_6_C carbide, (**c**) M_23_C_6_ carbide.

The base material microstructure is shown in Fig. 3. Therefore, alloy 625 grade I (soft annealed) has an amount of M(C, N) carbonitrides (Fig. 3a), M_6_C (Fig. 3b), and M_23_C_6_ carbides (Fig. 3c). In this context, M(C, N) carbonitrides were mainly located in the matrix, with the metal being Nb or Ti. In addition, M_6_C and M_23_C_6_ carbides, typically rich in Mo and Cr, were observed at isolated grain boundaries, as prior reported in^2,28^. In general, when etching with glyceregia, the matrix around M_6_C carbide is less etched than the matrix around M_23_C_6_ carbide, as shown in Fig. 3b,c. Figure 3c shows an M_23_C_6_ carbide (0.25 μm) at the grain boundary where some grooves around it can be observed, and thus neighboring metal-depleted zones may be more susceptible to intergranular corrosion. As these carbides are very small, the EDS result can be influenced by the interaction of the electron beam with alloy 625 matrix due to the beam diameter, which is larger than the carbide size. Furthermore^17^, also verified microstructural features as MC, M_6_C, and M_23_C_6_, suggesting that they evaluated a similar alloy condition.

In^17,19,28^, it has been indicated that Mo-depleted zones strongly influence the corrosion resistance, which can be understood by the pitting resistance equivalent number (PREN) calculated using Eq. (2). Thus, Mo-rich phases could result in lower local PREN in their depleted adjacent zones. In this work, as M_6_C and M_23_C_6_ carbides were observed in the soft annealed condition, and, since the latter group was mostly at grain boundaries, it seemed to have the main effect on sensitization and further intergranular corrosion.

2 $${\text{PREN }} = \, \% {\text{Cr }} + { 3}.{3}\left( {\% {\text{Mo }} + \, 0.{5}\% {\text{W}}} \right) \, + { 16}\% {\text{N}}$$

### Friction-stir-welded alloy 625

**Figure 4:**
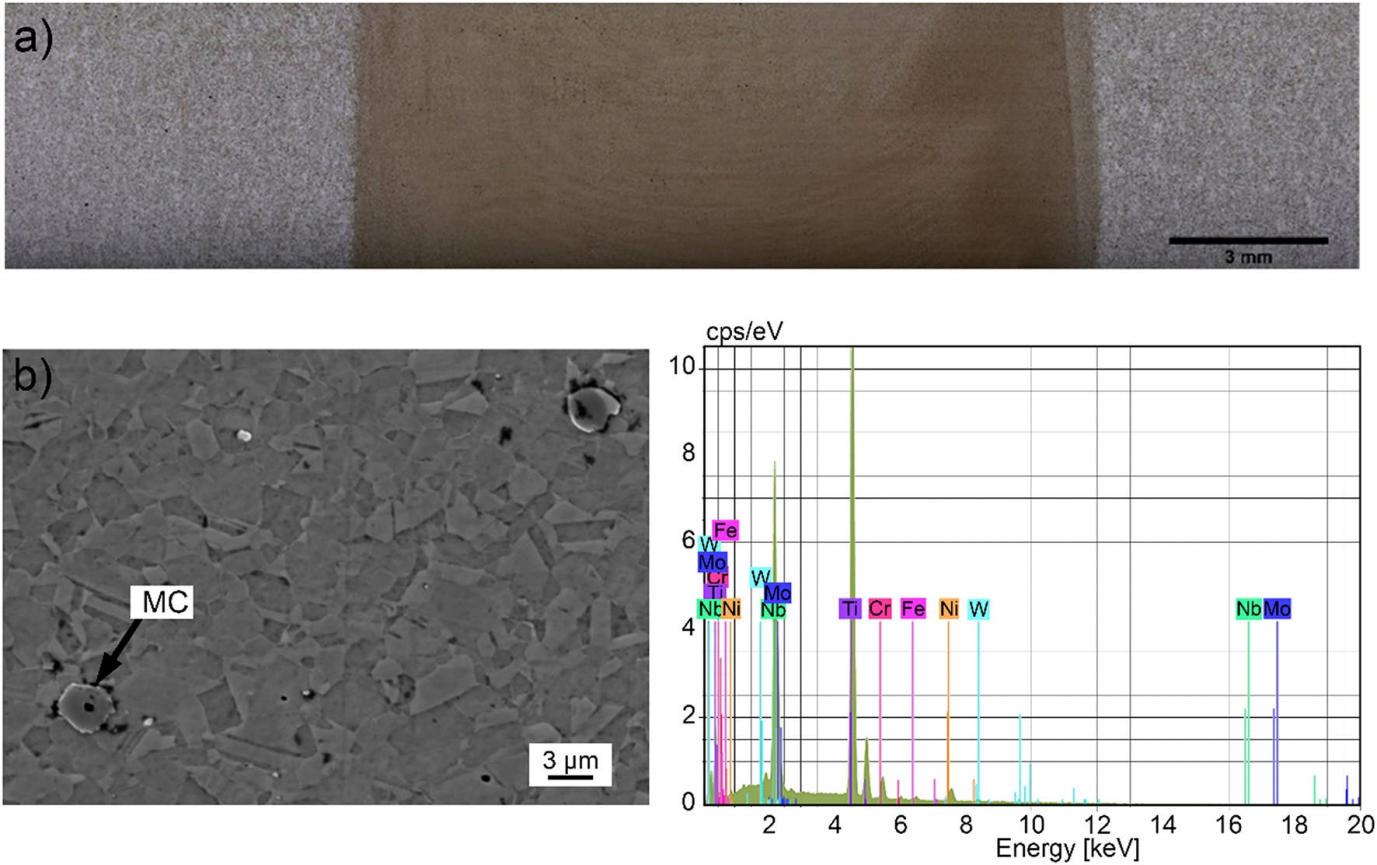
Friction-stir-welded alloy 625: (**a**) top surface macrostructure, (**b**) center of the stir zone.

The macrostructure on the top surface of friction-stir-welded alloy 625 is shown in Fig. 4a, where small-sized grains are verified through a darker microstructure (next detailed in Fig. 4b). Therefore, an MC type carbonitride (metal being Ti) was noted as well as a remarkable grain refinement of the austenite microstructure, which is typically accomplished in FSW of alloy 625^2,9^. In addition, no significant quantity of M_6_C and M_23_C_6_ carbides were observed by SEM imaging. Thus, it seems that most of the M_6_C and M_23_C_6_ carbides at grain boundaries (or even all) were fragmented and dissolved due to the stirring action of this severe deformation process (SDP). Furthermore, EDS analysis indicated tungsten (W) content that is not an alloying element of the alloy 625 base material and hence it came from the pcBN tool wear. In our recent work^24^, it was demonstrated that the average %W in the SZ (on the top surface) of this joint was 0.27%wt (lowest value among the FSW conditions evaluated), which represents minimal tool wear.

**Figure 5:**
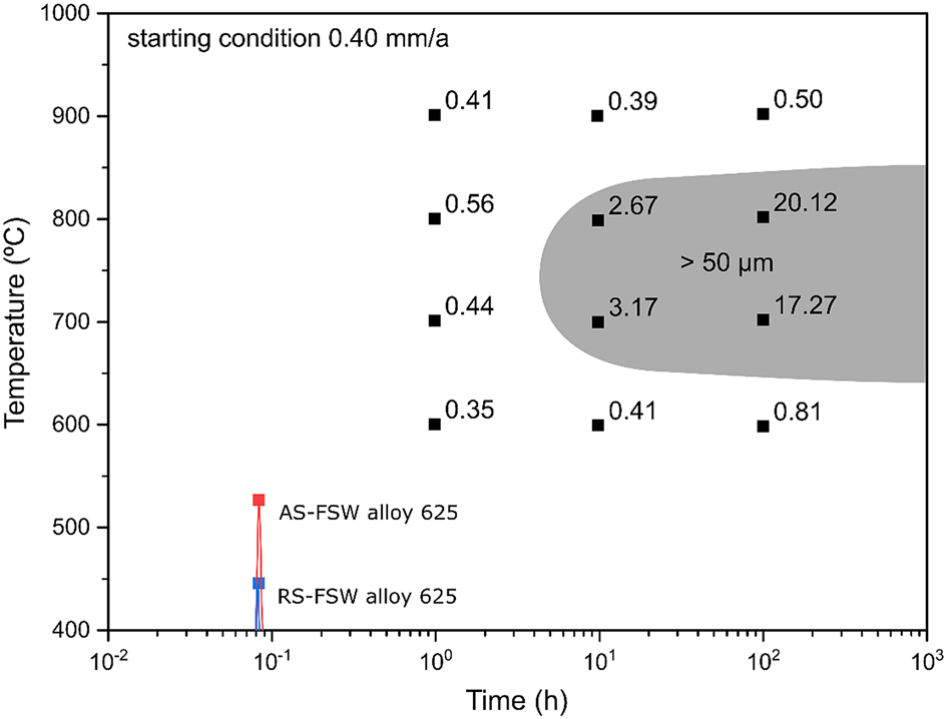
Time–temperature-sensitization diagram of alloy 625 where numbers indicate corrosion loss (mm/year) when tested according to ASTM G28 Method A (adapted from [^23^]), along with thermal cycles measured in FSW of alloy 625. AS refers to the advancing side, and RS to the retreating side.

Figure 5 presents the time–temperature-sensitization diagram of alloy 625 grade I (soft annealed) related to corrosion loss (mm/year) when tested according to ASTM G28 Method A^23^, along with the thermal cycles measured in the current investigation (temperatures at 15 mm distance from the weld centerline). With the soft annealed condition, sensitization of alloy 625, regarding the 50 µm criterion, could occur at around 750 °C after approximately 3 h^23^. In this work, the maximum temperature verified on the advancing side (AS) (513 °C) was higher than that on the RS (retreating side) (436 °C)^28^. Moreover, it is interesting to note that the FSW process would not lead to considerable sensitization in alloy 625 (temperatures reached were outside of the “C curve” for substantial sensitization). Otherwise, FSW tends to promote cleaner microstructure compared to the alloy 625 (grade I) base material, thus diminishing the susceptibility to sensitization. The cleaner microstructure in the SZ concerning the carbides at grain boundaries is a consequence of the recrystallization during FSW. Thus, the movement of recrystallization interfaces promoted the dissolution of M_23_C_6_ and M_6_C carbides.

**Figure 6:**
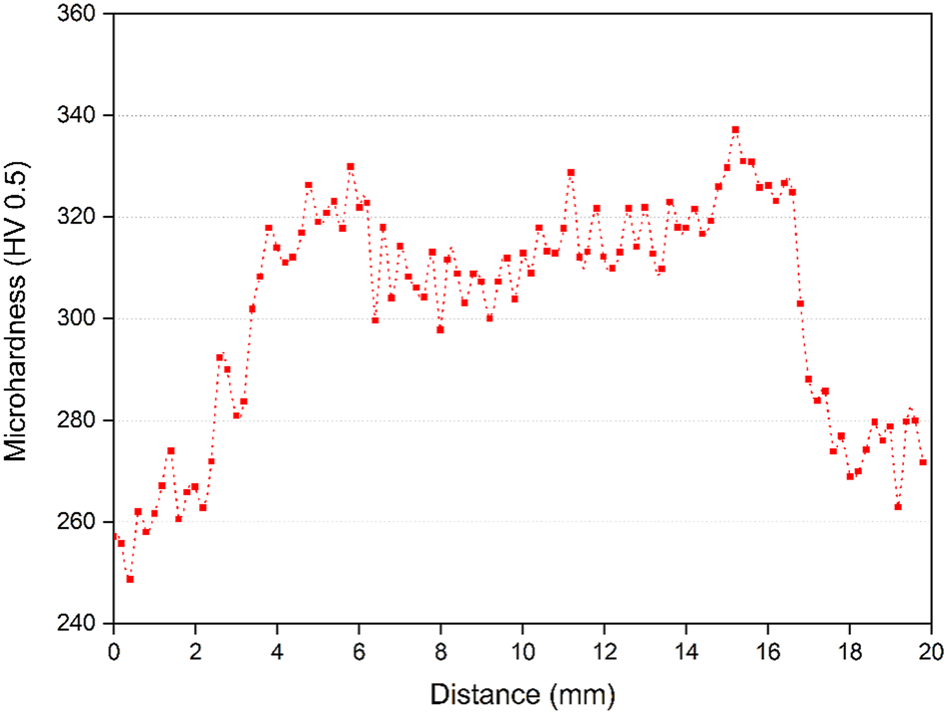
Vickers microhardness profile on the top surface of friction-stir-welded alloy 625.

The Vickers microhardness profile measured on the top surface can be seen in Fig. 6. Therefore, alloy 625 grade I (base material) had microhardness values ranging from 250 to around 280 HV. In the stir zone (SZ), microhardness values up to approximately 340 HV were achieved. Thus, the increased microhardness in the weld zone is commonly explained by finer grains obtained through FSW (clearly seen in Fig. 4). Recently, we suggested that an achievement of small-sized grains due to the FSW process can be related to the dynamic recrystallisation coming from the interaction between two variables (plastic flow and heat generated), which are sufficient to thus drive the recrystallization process^28^.

**Figure 7:**
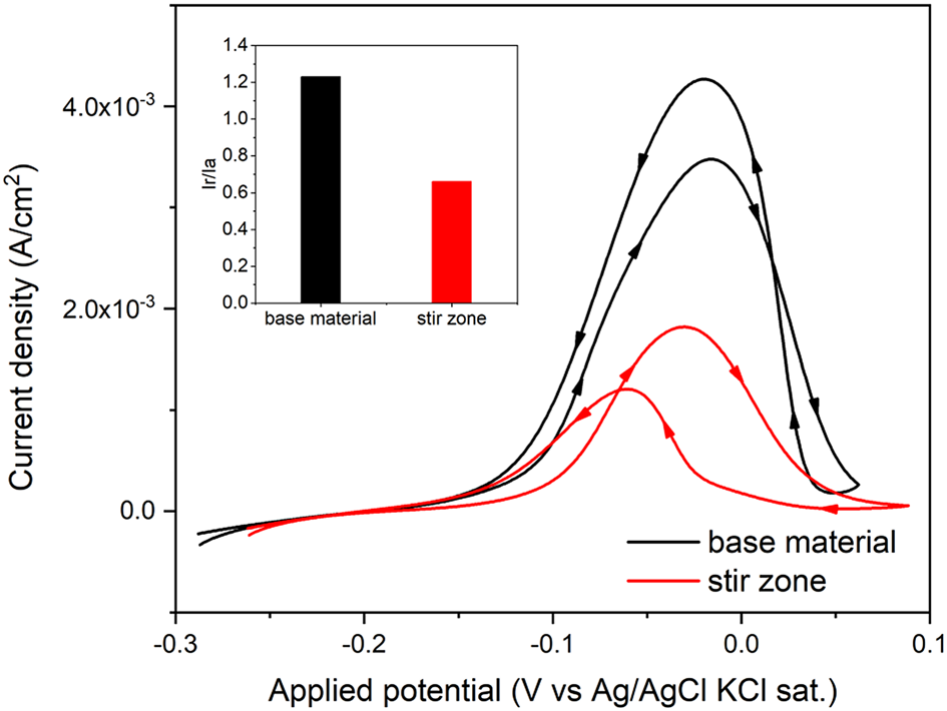
Double-loop electrochemical potentiokinetic reactivation test for the selected regions.

In Fig. 7, the differences between the peak activation current density (Ia) and the peak reactivation current density (Ir) indicate a distinct electrochemical behavior among the passivation and depassivation for each region evaluated. Moreover, a significant variation in the Ir/Ia ratio between the base material (BM) and the stir zone (SZ) was verified. Thus, it was noted that alloy 625 grade I soft annealed achieved a degree of sensitization of 1.23. On the other hand, FSW promoted a clean resulting microstructure concerning the carbides that would influence sensitization, a fact that caused a lower degree of sensitization (0.66) in SZ. This has good agreement with the findings shown in Figs. 4 and 5. Other studies reported similar outcomes, also indicating that FSW can be beneficial to mitigate the susceptibility to sensitization of distinct metals (AA 5083 aluminum alloy^30^ and 304 stainless steel^31^). The DL-EPR results indicate that although the grain refinement may reduce the intergranular corrosion resistance; the presence of M_23_C_6_ and M_6_C carbides is suggested to be more deleterious for the intergranular corrosion, resulting in an SZ with improved intergranular corrosion resistance.

The corrosion rate evaluated by the ASTM G28 Method A test is presented in Table 2, where both samples showed similar weight losses. Thus, the average corrosion rate was found to be of 0.4406 mm/year. According to^5^, the error of this test is ± 0.05 mm/year. In addition, some authors mentioned that the threshold for sensitization in the industry is 1 mm/year^13^, thus an acceptable friction-stir-welded alloy 625 was obtained. It is worth noting that authors^32^ showed that Inconel 625, produced by wire-arc additive manufacturing (WAAM), reached an average corrosion rate of 0.609 mm/year (slightly higher than that of the current work), which was then suggested to have an excellent resistance to intergranular corrosion. Furthermore, for Inconel 686, a nickel-based alloy that was processed by fusion welding processes such as gas metal-arc welding (GMAW) and gas tungsten-arc welding (GTAW), the corrosion rate was respectively 2.5 mm/year and 2.3 mm/year^33^. Overall, based on the above observations, a suitable welded joint of alloy 625 was produced by solid-state welding (FSW process).

**Table 2:** Assessment of susceptibility to intergranular corrosion of friction-stir-welded alloy 625.

Sample	Area (cm^2^)	Initial weight (g)	Final weight (g)	Weight loss	Corrosion rate (mm/year)
I	15.235	9.6966	9.6187	0.779	0.4422
II	16.373	11.0078	10.9247	0.0831	0.4390
				Average	0.4406

**Figure 8:**
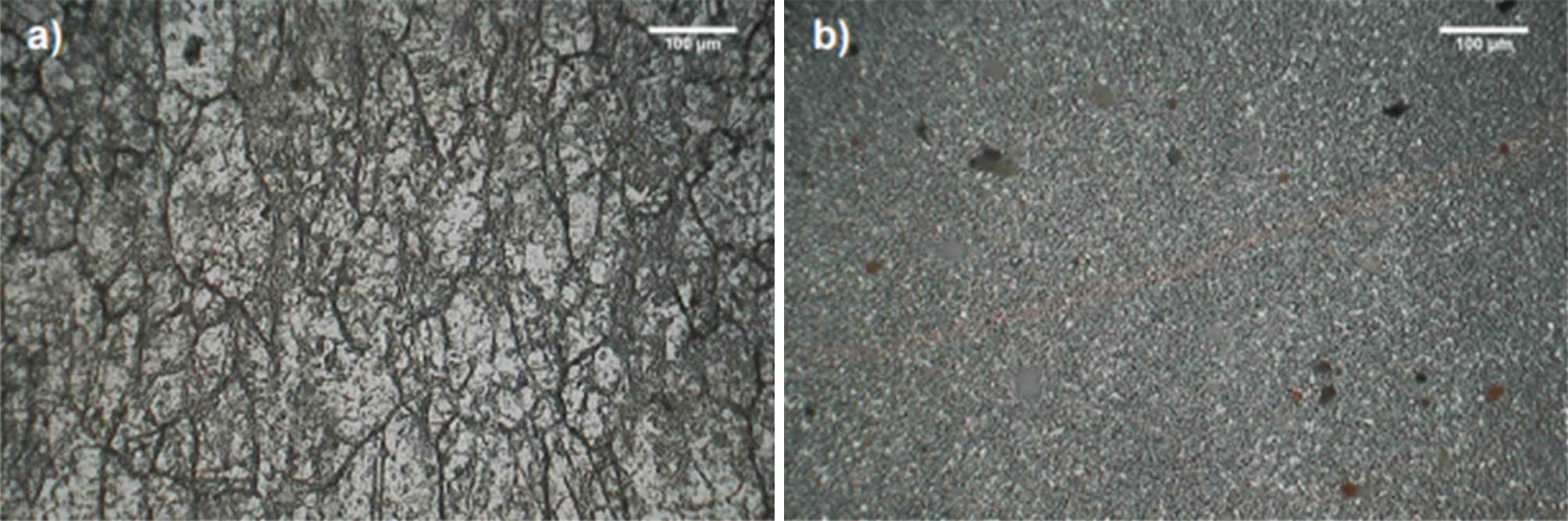
Micrographs after ASTM G28 Method A: (**a**) base material (BM), (**b**) stir zone (SZ).

Figure 8 shows the micrographs after using ASTM G28 Method A. As this assessment comprises the weight loss, and the BM had a certain amount of M_6_C and M_23_C_6_ at some grain boundaries, and these carbides are the most relevant to intergranular corrosion, it is assumed that the BM lost more weight than the SZ. Thus, it was noted that the BM (Fig. 8a) achieved a worse behavior than the SZ (Fig. 8b) regarding intergranular corrosion, which agrees well with the outcomes from DL-EPR test (Fig. 7). Therefore, the BM had the highest degree of sensitization and weight loss. In addition, in this test, grain dropping would take place if the alloy condition has a certain level of sensitization^19^, as here seen in the BM. In contrast, the SZ showed improved resistance to intergranular corrosion. Moreover, intergranular boundaries were not deeply corroded in the SZ compared with that of the BM. In other words, FSW thus mitigated the susceptibility to intergranular corrosion of alloy 625 grade I soft annealed. In a similar way, for FSW in AISI 304^34^, it was also revealed that the SZ had better corrosion properties than the BM.

## Conclusions

This work presented friction stir welding (FSW) for mitigating the susceptibility to intergranular corrosion of alloy 625 grade I (soft annealed). The results of the present investigation can be summarized as follows:

1. FSW led to the formation of small-sized grains and increased microhardness in the stir zone.
2. Double-loop electrochemical potentiokinetic reactivation (DL-EPR) tests showed that FSW decreased the degree of sensitization of alloy 625, which is mainly related to the absence of carbides at grain boundaries (verified by SEM) that would influence sensitization.
3. The mean corrosion rate verified with the intergranular corrosion ASTM G28 Method A was 0.4406 mm/year, which agrees with DL-EPR findings and suggests a good weld quality.
4. The presence of M_23_C_6_ and M_6_C carbides at grain boundaries is more deleterious for intergranular corrosion resistance. Therefore, the FSW process diminished the susceptibility to intergranular corrosion of alloy 625 grade I soft annealed. Furthermore, if a nickel-based alloy achieves a high level of sensitization due to fusion welding, manufacturing processes, or even service time–temperature conditions, it can be mitigated by using FSW.
